# Hospitalized Bacteremic Melioidosis in Rural Thailand: 2009–2013

**DOI:** 10.4269/ajtmh.17-0402

**Published:** 2018-04-02

**Authors:** Anchalee Jatapai, Christopher J. Gregory, Somsak Thamthitiwat, Kittisak Tanwisaid, Saithip Bhengsri, Henry C. Baggett, Ornuma Sangwichian, Possawat Jorakate, John R. MacArthur

**Affiliations:** 1 Global Disease Detection Regional Center, Thailand Ministry of Public Health–U.S. Centers for Disease Control and Prevention Collaboration, Nonthaburi, Thailand;; 2 Division of Global Health Protection, Center for Global Health, Centers for Disease Control and Prevention, Atlanta, Georgia;; 3 Nakhon Phanom Provincial Hospital, Nakhon Phanom, Thailand

## Abstract

Melioidosis incidence and mortality have reportedly been increasing in endemic areas of Thailand, but little population-based data on culture-confirmed *Burkholderia pseudomallei* infections exist. We provide updated estimates of melioidosis bacteremia incidence and in-hospital mortality rate using integration of two population-based surveillance databases in Nakhon Phanom, Thailand, since automated blood culture became available in 2005. From 2009 to 2013, 564 hospitalized bacteremic melioidosis patients were identified. The annual incidence of bacteremic melioidosis ranged from 14 to 17 per 100,000 persons, and average population mortality rate was 2 per 100,000 persons per year. In-hospital mortality rate declined nonsignificantly from 15% (15/102) to 13% (15/118). Of 313 (56%) bacteremic melioidosis patients who met criteria for acute lower respiratory infection and were included in the hospital-based pneumonia surveillance system, 65% (202/313) had a chest radiograph performed within 48 hours of admission; 46% (92/202) showed radiographic evidence of pneumonia. Annual incidence of bacteremic melioidosis with pneumonia was 2.4 per 100,000 persons (95% confidence intervals; 1.9–2.9). In-hospital death was more likely among bacteremic melioidosis patients with pneumonia (34%; 20/59) compared with non-pneumonia patients (18%; 59/321) (*P-*value = 0.007). The overall mortality could have been as high as 46% (257/564) if patients with poor clinical condition at the time of discharge had died. The continued high incidence of bacteremic melioidosis, pneumonia, and deaths in an endemic area highlights the need for early diagnosis and treatment and additional interventions for the prevention and control for melioidosis.

## INTRODUCTION

Melioidosis is a severe infection caused by the highly pathogenic Gram-negative bacillus, *Burkholderia pseudomallei,* found in soil and water throughout Southeast Asia.^1^ This organism is an important cause of community-acquired sepsis and pneumonia in tropical areas with infection resulting from contact with contaminated soil or water via percutaneous inoculation, wound contamination, ingestion, or aerosol inhalation.^1,2^ Melioidosis affects people living in and traveling to endemic areas with increasing numbers of patients being reported in northeastern Thailand, northern Australia, Singapore, Malaysia, and parts of Africa.^3–9^

Septicemic melioidosis is a major cause of morbidity and the third most frequent cause of death from infectious disease in northeastern Thailand,^10^ and its incidence is highest in the northeastern region with recent estimates of 12.7,^10^ 14.9,^9^ and 24.1^11^ infections per 100,000 persons per year. Confirming *B. pseudomallei* infection by using culture requires experienced microbiology laboratory staff with specialized knowledge and techniques, which has contributed to the limited population-based data on culture-confirmed *B. pseudomallei* infections.

Effective treatment of melioidosis requires at least 10–14 days of parenteral treatment and an additional 12–20 weeks of oral antibiotic treatment, and *B. pseudomallei* is resistant to usual first-line drugs for suspected sepsis.^12^ Even with appropriate therapy the case fatality rate (CFR) of melioidosis remains high, ranging from 37% to 76% in Thailand.^12–15^ The CFR may vary based on mode of acquisition, severity of disease, and access to health care. In endemic areas, community-acquired pneumonia is a common presentation of melioidosis.^16^ In northern Australia, overall CFR of melioidosis was 14% and up to 45% of deaths were in patients with septicemia complicated by pneumonia,^17^ despite the receipt of appropriate treatment.^2^

To date, there is no licensed human vaccine to protect people exposed to *B. pseudomallei* during their daily activities such as working in rice paddies, military training, or touring endemic areas. Diagnostic tools and therapeutic resources are also very limited in most endemic areas. It is therefore essential to understand the trends and outcome of bacteremic melioidosis to better guide clinical and public health management, to more rapidly detect, respond to, and control melioidosis at its source, and thereby enhance global health security. The objectives of this population-based investigation were to update melioidosis disease burden estimates, outcome, and drug susceptibility patterns following the introduction of enhanced microbiological diagnostic capacity in Nakhon Phanom province, Thailand.

## MATERIALS AND METHODS

### Study design and study population.

During 2003–2014, the U.S. Centers for Disease Control and Prevention (CDC) collaborated with the Thailand Ministry of Public Heath to conduct enhanced population-based surveillance for pneumonia hospitalizations in Nakhon Phanom province. Nakhon Phanom is located in upper northeastern Thailand and borders the Lao People’s Democratic Republic, with an estimated population ∼700,000 people, 58% of them farmers.^18^ In 2005, bloodstream infection (BSI) surveillance was initiated with the implementation of an automated blood culture system and conducted in all hospitals in the province including the provincial hospital (∼300 beds) and 11 district or military hospitals (10–140 beds). These surveillance systems underwent human subjects review at the CDC, and were determined to be routine public health surveillance and not require review by an institutional review board.

### Case definitions.

**Figure 1.**
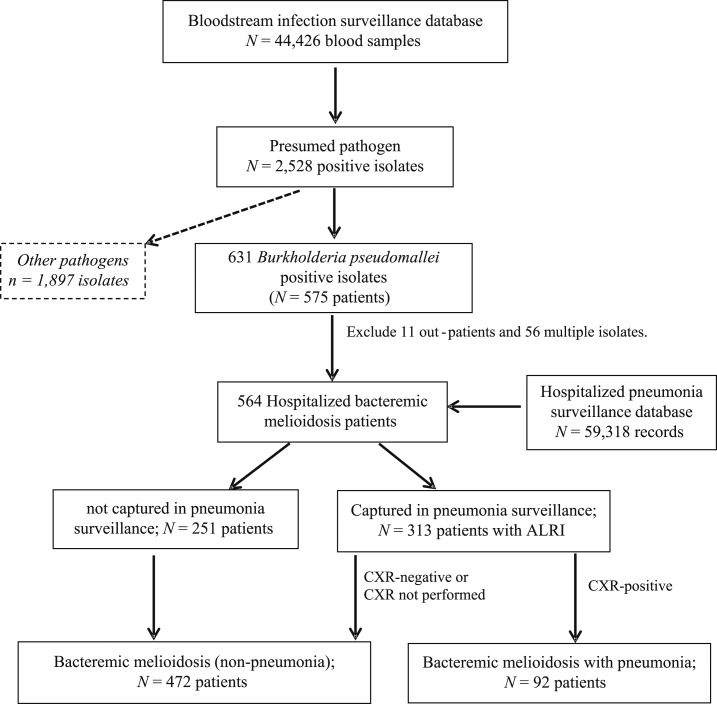
Flow diagram of melioidosis patients from the integration of bloodstream infection and hospitalized pneumonia surveillance in Nakhon Phanom, Thailand; 2009–2013. Note: chest radiograph (CXR) positive, defined as radiographic evidence of pneumonia (consolidation and/or other infiltrates).

We defined a case of “bacteremic melioidosis” as an isolation of *B. pseudomallei* from a blood culture in a hospitalized resident of Nakhon Phanom during January 2009 to December 2013. The cases were identified through BSI surveillance. For patients with *B. pseudomallei* identified on multiple cultures, regardless of time between cultures, only the first instance was included for analysis. The parallel pneumonia surveillance system in Nakhon Phanom was used to identify bacteremic melioidosis patients with pneumonia.^19–21^ This system defined a case of acute lower respiratory infection (ALRI) as a person hospitalized with ≥ 1 sign/symptom of acute infection (reported fever, measured temperature > 38.2°C or < 35°C, chills) or an abnormal white blood cell count (> 11,000 or < 3,000 cells/mL) and ≥ 1 respiratory sign or symptoms (abnormal breath, tachypnea, cough, chest pain, hemoptysis, sputum production, or dyspnea). Patients meeting the ALRI case definition who had a chest radiograph (CXR) performed per clinician discretion within 48 hours post-admission with infiltrate consistent with that of CXR-pneumonia were defined as “bacteremic melioidosis with pneumonia” (Figure 1). Chest radiograph–confirmed pneumonia was determined by a panel of radiologists in Bangkok using standard criteria as previously described^22,23^ or by treating clinicians. Detailed clinical and demographic information was available for patients who met inclusion criteria for pneumonia surveillance. In-hospital outcomes were determined based on information in the medical record. Patients were categorized as having poor clinical condition if discharge status was recorded as “not improved” and discharge type as “by transfer,” “escape” (they checked themselves out), and “against advice” (suggesting the family brought them home in moribund condition).^19–21^

### Specimen and laboratory methods.

All patients with suspected sepsis had blood culture collected per clinical discretion. Blood cultures collected at community hospitals were transported at 15–30°C for centralized testing at the Nakhon Phanom provincial hospital microbiology laboratory within 24 hours.^24^ Those specimens were incubated in the BactT/ALERT^®^ 3D automated blood culture system (BioMérieux, Marcy-l’Étoile, France). Bottles which signaled positive growth were subcultured onto sheep blood, chocolate, and MacConkey agar plates and then incubated for further gram staining, biochemical tests, and commercial bacterial identification strips (API strips; BioMérieux) to identify positive bacterial cultures^25^ including *B. pseudomallei*. From 2010 to 2013, antibiotic susceptibility testing was performed using minimum inhibitory concentration strips (E-test; BioMérieux) for ceftazidime and trimethoprim/sulfamethoxazole, antibiotics typically used to treat melioidosis patients in this province. Isolates were classified as resistant or susceptible using resistance breakpoints per Clinical and Laboratory Standards Institute recommendations.^26^

### Statistical analyses.

We estimated overall, gender-, and age-specific incidence rates using population denominators from the National Economic and Social Development Board of Thailand.^27^ Exact 95% confidence intervals (CI) were calculated based on a Poisson distribution. To evaluate for possible trends, we performed linear regression with robust variance and nonparametric tests to assess potential changes in incidence and in-hospital mortality rate over time. To account for possible underestimation of *B. pseudomallei* mortality due to discharge of patients still in poor clinical condition, we performed a sensitivity analysis using a “worst-case” approach assuming that up to 100% of patients discharged in poor clinical condition died after leaving the hospital compared with a baseline analysis, which excluded all patients with poor discharge outcome in mortality calculations. A *P-*value < 0.05 was considered significant. All analyses were performed using STATA version 12.0 (StataCorp LP, College Station, TX).

## RESULTS

A total of 44,426 blood culture samples were collected from patients during January 2009 to December 2013 in Nakhon Phanom province. Of these, 2,528 (5.7%) cultures from 2,031 patients had a presumed pathogen isolated. A total of 631 *B. pseudomallei* isolates from 575 patients were identified; after excluding 11 outpatients and 56 patients with multiple positive cultures, 564 hospitalized bacteremic melioidosis patients were included in the analysis. Among these 564 patients with bacteremic melioidosis, 313 (56%) had ALRI and were also captured by the pneumonia surveillance system (Figure 1).

### Incidence of hospitalized bacteremic melioidosis.

**Figure 2.**
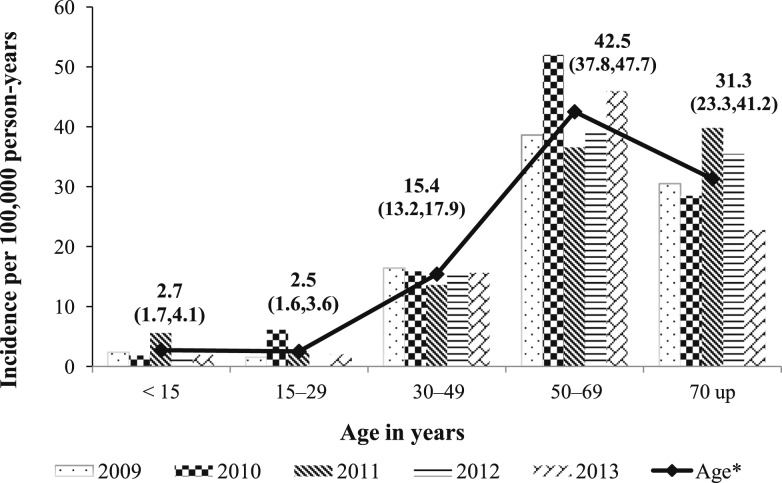
Incidence rate of hospitalized bacteremic melioidosis by year and age, Nakhon Phanom, Thailand; 2009–2013.

The overall incidence rate of bacteremic melioidosis hospitalizations in Nakhon Phanom, Thailand, during 2009–2013 was 14.9 per 100,000 persons per year. Annual incidence ranged from 13.7 per 100,000 persons in 2009 to 17.2 per 100,000 persons in 2010 (Table 1). Males comprised 60% of hospitalized bacteremic melioidosis patients and the overall incidence rate was higher in males than females (Table 1). During the investigation period, bacteremic melioidosis occurred in patients aged 1 to 93 years old; median age was 53 years (interquartile range 43–62 years), and 22 (4%) bacteremic melioidosis patients were younger than 15 years old. The highest incidence was observed among patients aged 55–64 years at 46.9 per 100,000 persons per year (95% CI; 39.9–54.8) (Table 1). Age-specific incidence rate by year is shown in Figure 2. The number of bacteremic melioidosis cases was significantly higher in the rainy season, 304 cases in July–October with a peak in August (19%) compared with the dry season, 121 cases in March–June (*P-*value = 0.037).

**Table 1:** Incidence rate and in-hospital mortality rate of hospitalized bacteremic melioidosis, Nakhon Phanom, Thailand; 2009–2013

Year, characteristics	Population	Bacteremic melioidosis patients	In-hospital deaths	IR	95% CI	In-hospital mortality rate (%)
2009	746,655	102	15	13.7	(11.1–16.6)	14.7
2010	751,251	129	20	17.2	(14.3–20.4)	15.5
2011	754,931	108	14	14.3	(11.7–17.3)	13.0
2012	758,388	107	15	14.1	(11.6–17.1)	14.0
2013	761,623	118	15	15.5	(12.8–18.6)	12.7
Overall 5 years	3,772,848	564	79	14.9	(13.7–16.2)	14.0
Gender						
Female	1,889,272	226	31	12	(10.5–13.6)	13.7
Male	1,883,576	338	48	17.9	(16.1–20.0)	14.2
Age group (year)						
< 5	240,639	10	3	4.2	(2.0–7.6)	30.0
5–14	563,215	12	2	2.1	(1.1–3.7)	16.7
15–24	657,765	11	2	1.7	(0.8–3.0)	18.2
25–34	612,517	31	4	5.1	(3.4–7.2)	12.9
35–44	581,016	95	10	16.4	(13.2–20.0)	10.5
45–54	510,421	145	19	28.4	(24.0–33.4)	13.1
55–64	336,942	158	26	46.9	(39.9–54.8)	16.5
65–74	182,009	72	8	39.6	(31.0–49.8)	11.1
> 75	88,323	30	5	34	(22.9–48.5)	16.7

CI = confidence intervals; IR = incidence rate per 100,000 persons per year.

Of 313 bacteremic melioidosis patients with ALRI, 202 (65%) had a CXR performed within 48 hours after admission, and 92 (29%) had radiographic evidence of pneumonia. Therefore, among all 564 bacteremic melioidosis case patients, 92 (16%) had pneumonia. The incidence rate of hospitalized bacteremic melioidosis with pneumonia was 2.4 per 100,000 persons per year (95% CI; 1.9–2.9). Among bacteremic melioidosis patients with pneumonia, the most common clinical characteristics were rales or crepitation (84%), fever > 38°C (74%), cough (66%), and dyspnea (66%); 74% required oxygen supplementation.

Among the 564 hospitalized bacteremic melioidosis patients, 52% (294) were admitted in community hospitals and 48% (270) in a provincial hospital. Patients admitted in the provincial hospital were more likely to have severe respiratory illness as demonstrated by 40% requiring intubation (54/138) compared with 11% (20/175) of patients admitted to community hospitals, *P*-value < 0.001. We observed differences in the prevalence of the following clinical measures between bacteremic melioidosis patients with ALRI admitted to the provincial hospital compared with those admitted to community hospitals: dyspnea (62% versus 28%), tachypnea (43% versus 21%), wheezing (24% versus 43%), confusion (21% versus 9%), oxygen therapy (64% versus 49%), intubation (39% versus 10%), abnormal white blood cell count (60% versus 43%), and comorbidity of renal disease (41% versus 26%) and liver disease (19% versus 3%); *P*-value < 0.05.

### Deaths among hospitalized bacteremic melioidosis.

**Figure 3.**
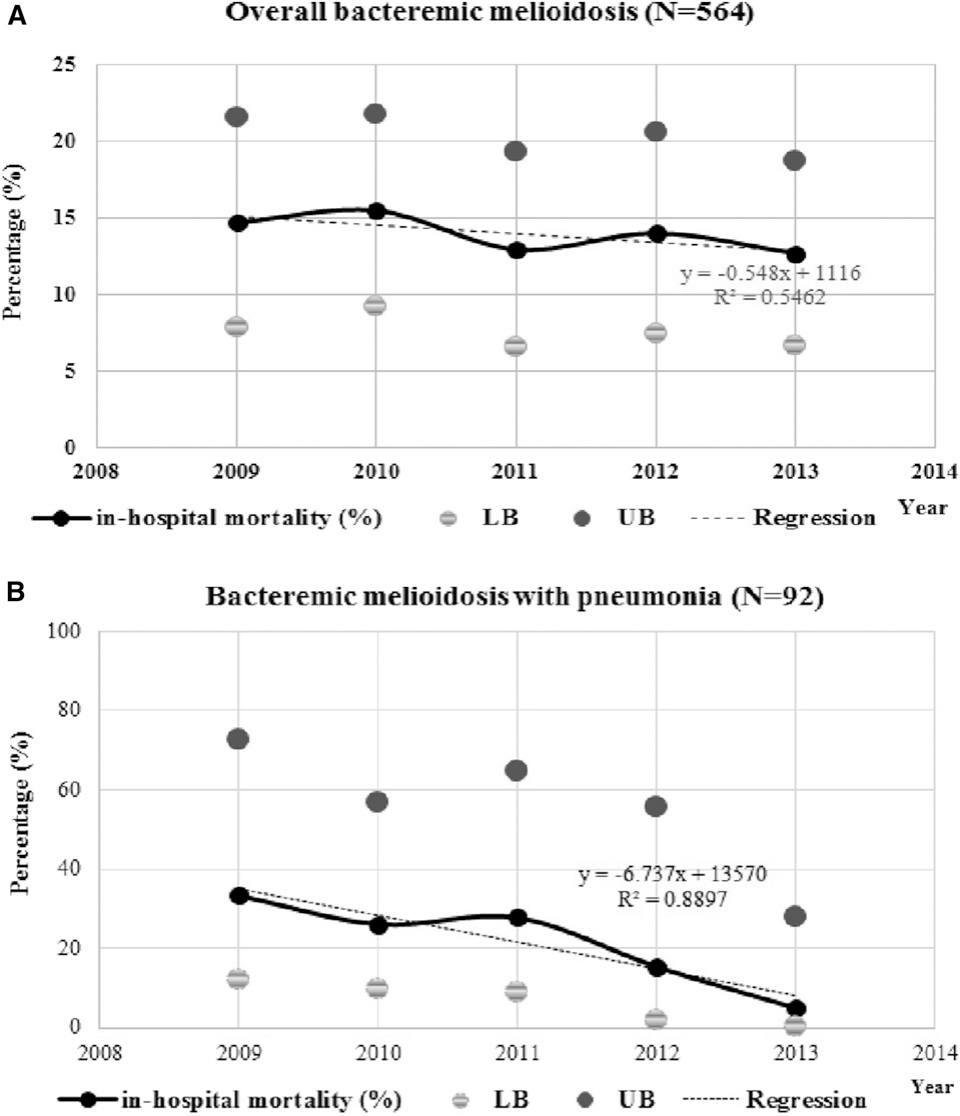
In-hospital mortality rate with upper bound (UB) and lower bound (LB) of 95% confidence interval. **(A)** Bacteremic melioidosis with linear regression for trend (*P*-value = 0.056) and **(B)** bacteremic melioidosis with pneumonia (*P*-value = 0.006) in Nakhon Phanom, Thailand.

In-hospital death occurred in 79 (14%) of 564 bacteremic melioidosis patients. Most (50/79) deaths occurred within 2 days after admission. Median duration of hospital stay before death was 2 days with a range of 1–21 days. The in-hospital fatality rate declined from 15% in 2009 to 13% in 2013 (slope of regression; −0.5%, 95% CI; −1.1–0.03%, Figure 3A). The in-hospital fatality rate among bacteremic melioidosis patients was highest in November at 19% (8/43) compared with the month with the highest number of cases in August 13% (14/106). The overall population mortality rate was 2.1 per 100,000 persons per year (95% CI; 1.7–2.6) and slightly decreased from 2.7 per 100,000 persons per year (95% CI; 1.6–4.1) in 2010 to 2.0 per 100,000 persons per year (95% CI; 1.1–3.3) in 2013. The observed in-hospital fatality rate among bacteremic melioidosis pneumonia patients significantly decreased from 33% (6/18) in 2009–5% (1/20) in 2013 (*P*-value = 0.006) (Figure 3B). Thirty-nine (42%) melioidosis pneumonia patients were intubated and mechanically ventilated, of whom 51% (20/39) died in the hospital and 41% (16/39) were discharged in poor condition. In-hospital mortality rate among patients admitted to the provincial hospital was higher than that among cases admitted to community hospitals (33% [67/206] versus 7% [12/173], *P*-value < 0.001).

Potentially poor clinical outcome at the time of discharge was observed at 32% (178/564) of bacteremic melioidosis patients, including 139 (25%) who transferred to another hospital, 22 (4%) discharged against advice, and 17 (3%) escaped (Table 2). Outcome status was listed as poor condition in 47% (34/72) of bacteremic melioidosis patients with pneumonia compared with 35% (144/413) of non-pneumonia cases (*P*-value = 0.045). Given the high percentage of patients with potentially poor clinical outcome at the time of discharge, we performed a sensitivity analysis to account for a range of overall death rate among patients discharged in poor condition. The overall death rate ranged from 14% to 46%; 22% to 59% among patients with pneumonia; and 13% to 43% among non-pneumonia patients. Patients who had poor clinical outcome at discharge were more likely to have dyspnea (48% versus 31%), abnormal breath (40% versus 24%), tachypnea (36% versus 23%), oxygen therapy (71% versus 37%), intubation (30% versus 4%), and confusion (19% versus 8%) compared with survivors; *P*-value < 0.05.

**Table 2:** Comparison of outcomes between hospitalized bacteremic melioidosis patients with and without pneumonia, Nakhon Phanom, Thailand; 2009–2013

Outcome	Hospitalized bacteremic melioidosis patients		
	Total; *N* = 564, *n* (%)	Pneumonia	Non-pneumonia*
		*N* = 92, *n* (%)	*N* = 472, *n* (%)
Survived	307 (54.4)	38 (41.3)	269 (57.0)
In-hospital deaths	79 (14.0)	20 (21.7)	59 (12.5)
Poor discharge condition†	178 (31.6)	34 (37.0)	144 (30.5)
Baseline analysis (%)			
Overall mortality rate	79 (26.2)	20 (52.6)	59 (22.4)
Sensitivity analysis (%)			
Minimal overall mortality rate‡	79 (14.0)	20 (21.7)	59 (12.5)
Moderate overall mortality rate§	124 (22.0)	29 (31.0)	95 (20.1)
High overall mortality rate‖	168 (24.1)	37 (40.2)	131 (27.8)
Maximum overall mortality rate¶	257 (45.6)	54 (58.7)	203 (43.0)

* Including chest radiograph (CXR) negative, CXR not performed, and no CXR information.

† Poor discharge condition of patients defined as documented discharge type of transfer, escape, or discharge against advice.

‡ No poor discharge status patients assumed to die.

§ Assume 25% of poor discharge status patients died.

‖ Assume 50% of poor discharge status patients died.

¶ Assume 100% of poor discharge status patients died.

### Drug susceptibility.

Among 513 *B. pseudomallei* isolates tested since 2010, 99.8% were susceptible to ceftazidime and 95.8% were susceptible to trimethoprim/sulfamethoxazole. No clear trend in resistance patterns during the study period was observed.

## DISCUSSION

Our enhanced population-based surveillance provides updated estimates of the incidence and in-hospital mortality rate of bacteremic melioidosis among hospitalized patients in Nakhon Phanom, Thailand, during 2009–2013. The estimated average annual incidence of bacteremic melioidosis hospitalizations was 14.9 per 100,000 persons, very similar to our previous estimates from 2006 to 2008,^9^ and slightly higher than a hospital-based study of melioidosis in northeastern Thailand from 1997 to 2006 (12.7 per 100,000 populations).^10^ Overall incidence showed variability between years from 13 to 17 per 100,000 persons per year with a nonsignificant decrease in in-hospital mortality rate from 2009 to 2013.

During 2009–2013, our incidence rate is still higher than country reported melioidosis morbidity rate from Thailand’s national surveillance system.^28^ During our investigation period, the national melioidosis morbidity rate ranged from 2.13 to 6.13 per 100,000 population with mortality rate less than 1%. This likely substantially underestimates the number of melioidosis cases and deaths in Thailand given the passive nature of the national surveillance system. Our investigation’s report showed the advantage of the integration of laboratory information and clinical information from hospitalized pneumonia surveillance to monitor melioidosis in this province. The ideal epidemiological system for monitoring infectious diseases morbidity and mortality would connect laboratory information, clinical information, and death registry data in the integrated reporting mechanism. The high observed incidence among patients aged 55–64 years may be due to this age group likely having higher prevalence of comorbid conditions known to be risk factors for melioidosis^17^ and having greater occupational exposures (e.g., rice farming).^29^ As noted in the recent melioidosis global burden of disease estimates, the true burden of this disease is likely still underestimated even in endemic areas.^30^ There was a marked temporal clustering of hospitalized bacteremic melioidosis patients during the rainy season from July through October similar to what has been reported elsewhere in Thailand and other endemic areas.^4,9,10,31^

The slight observed decrease in in-hospital mortality rate from 2009 to 2013 could have resulted from detection of more (and possibly less severe) cases through improved blood culturing practices or from improved recognition and awareness of the clinician for treating suspected melioidosis patients, especially during the rainy season since 2006. The in-hospital death of bacteremic melioidosis after installing the automated blood culture system decreased from 29% in 2006 to 21% in 2008^9^ and continued to decrease to 13% in 2013. Previously, the number of in-hospital deaths of hospitalized melioidosis cases in Nakhon Phanom was reported as high as 56% in 2003 and 34% in 2005.^32^ Given the observed frequency of patients being discharged in poor clinical condition noted mortality could range from a low of 14% when limited to in-hospital deaths to 46% if all patients with poor clinical condition at the time of discharge subsequently died. Unfortunately, our database did not obtain the Thai identification number for tracking out-of-hospital deaths through the national death registry database. Although limited by a low number of patients per month, the in-hospital mortality rate was noted to be highest in November during the cool season, which could suggest lower clinician awareness compared with the rainy season and may indicate a need for continued sensitization of clinicians during non-rainy periods of the year when incidence is lower. Clinicians in endemic regions should have a high index of suspicion for *B. pseudomallei* infection throughout the year and empirical septicemia treatment guidelines should include antibiotics active against *B. pseudomallei*.^12,17^ This is particularly true for patients in the highest risk age-groups (patients aged more than 50 years), those with previously identified risk factors for *B. pseudomallei* infection such as diabetes mellitus and renal insufficiency, and those groups with likely intense environmental exposure, such as rice farmers and other agricultural workers. During the 5-year analysis period, in-hospital mortality rate was higher among melioidosis patients with pneumonia, which is consistent with recent studies in other locations.^2,10^ Although limited by the small number of cases annually, the observed decline in in-hospital mortality rates among patients with melioidosis pneumonia deserves further investigation and could relate to greater clinician awareness of melioidosis among severely ill patients with pneumonia.

There were several limitations to our analyses. First, our investigation underestimated the overall incidence and burden of melioidosis in the province. We did not have information on outpatients or non-bacteremic melioidosis patients, and previous studies have shown that only around 50–60% of melioidosis patients in northeastern Thailand are bacteremic.^1^ In addition, the sensitivity of blood culture for diagnosis may be as low as 60%,^33^ so additional testing modalities would likely have identified more case patients. Second, we did not have information on outcome after hospital discharge, and more than a third of surviving patients had their discharge status listed as poor. Not including out-of-hospital outcomes likely led to an underestimation of overall mortality rate. Third, more than 40% of bacteremic melioidosis cases lacked details on clinical characteristics and antibiotic treatment, limiting the ability to assess overall disease severity and the impact of treatment on outcome among hospitalized bacteremic melioidosis patients.

The burden of bacteremic melioidosis in Nakhon Phanom remains high despite the reduction in in-hospital case fatality seen since the introduction of automated blood culture capacity in the province in 2005. The continued high incidence of bacteremic melioidosis, pneumonia, and associated deaths in an endemic area is concerning given increasing numbers of persons with high-risk conditions such as diabetes, the incidence of which increased from 658 to 894 per 100,000 persons from 2009 to 2013 in Nakhon Phanom province.^34^ Therefore, maintaining sensitive BSI surveillance, which provides a consistent and comparable method to monitor and track trends in serious invasive infections, is vital to guide the development of affordable strategies to reduce morbidity and mortality. Such surveillance activities are also important for rapidly detecting, responding to, and controlling this public health problem, and thereby contributing to global health security. We encourage clinicians working in *B. pseudomallei* endemic areas to investigate potential melioidosis patients using standardized, validated microbiological techniques together with CXR, especially for patients presenting with sepsis or acute respiratory symptom in the rainy season. Consistent application of this approach will optimize the opportunity for early diagnosis and targeted treatment to improve outcomes. Increased availability of tests with faster turnaround times than conventional blood culture including sensitive, point-of-care tests or use of advanced molecular methods, may allow for earlier provision of life-saving treatment. Promising options, including PCR, latex agglutination, and lateral flow assays have been recently developed, and evaluations of these tests in the clinical setting are underway.^35^ In the absence of an approved human vaccine, the identification of alternative prevention measures for melioidosis are urgently needed in high-burden areas, especially for known high-risk groups such as patients with diabetes, renal failure patients, and older adults.
